# Phytochemical Constituents from *Cercidiphyllum japonicum* Exhibit Bioactive Potential Against Skin Aging and Inflammation in Human Dermal Fibroblasts

**DOI:** 10.3390/cimb47080631

**Published:** 2025-08-07

**Authors:** Minseo Kang, Sanghyun Lee, Dae Sik Jang, Sullim Lee, Daeyoung Kim

**Affiliations:** 1 Department of Life Science, College of Bio-Nano Technology, Gachon University, Seongnam 13120, Republic of Korea; 2 Department of Plant Science and Technology, Chung-Ang University, Anseong 17546, Republic of Korea; 3 Natural Product Institute of Science and Technology, Anseong 17546, Republic of Korea; 4 College of Pharmacy, Kyung Hee University, 26 Kyungheedae-ro, Dongdaemun-gu, Seoul 02453, Republic of Korea

**Keywords:** *Cercidiphyllum japonicum*, ellagic acid, skin aging, oxidative stress, inflammation

## Abstract

With increasing interest in natural therapeutic strategies for skin aging, plant-derived compounds have gained attention for their potential to protect against oxidative stress and inflammation. In this study, we investigated the anti-aging and anti-inflammatory effects of flavonoids isolated from *Cercidiphyllum japonicum* using a tumor necrosis factor-alpha (TNF-α)-stimulated normal human dermal fibroblast (NHDF) model. The aerial parts of *C. japonicum* were extracted and analyzed by high-performance liquid chromatography (HPLC), leading to the identification of four major compounds: maltol, chlorogenic acid, ellagic acid, and quercitrin. Each compound was evaluated for its antioxidant and anti-aging activities in TNF-α-stimulated NHDFs. Among them, ellagic acid exhibited the most potent biological activity and was selected for further mechanistic analysis. Ellagic acid significantly suppressed intracellular reactive oxygen species (ROS) generation and matrix metalloproteinase-1 (MMP-1) secretion (both *p* < 0.001), while markedly increasing type I procollagen production (*p* < 0.01). Mechanistic studies demonstrated that ellagic acid inhibited TNF-α-induced phosphorylation of mitogen-activated protein kinases (MAPKs), downregulated cyclooxygenase-2 (COX-2), and upregulated heme oxygenase-1 (HO-1), a key antioxidant enzyme. Additionally, ellagic acid attenuated the mRNA expression of inflammatory cytokines, including interleukin-6 (IL-6) and interleukin-8 (IL-8), indicating its broad modulatory effects on oxidative and inflammatory pathways. Collectively, these findings suggest that ellagic acid is a promising plant-derived bioactive compound with strong antioxidant and anti-inflammatory properties, offering potential as a therapeutic agent for the prevention and treatment of skin aging.

## 1. Introduction

The skin is the largest organ of the human body, accounting for approximately 15% of total body weight, and serves as the primary interface between the body and the external environment. It functions not only as a physical barrier but also as an immunological and sensory organ, playing a critical role in maintaining homeostasis and protecting internal organs from various environmental insults, such as microbial invasion, ultraviolet (UV) radiation, air pollution, and chemical irritants [1]. Anatomically, the skin consists of a multilayered epidermis and an underlying dermis, which is rich in fibroblasts, blood vessels, and extracellular matrix (ECM) components [2,3]. The ECM is a key structural and functional component of the dermis, composed primarily of collagen, elastin, hyaluronic acid, and proteoglycans. These molecules maintain the skin’s strength, elasticity, hydration, and integrity. However, with advancing age, both the quantity and quality of ECM components decline due to intrinsic and extrinsic factors, leading to characteristic signs of skin aging such as fine lines, wrinkles, dryness, and reduced elasticity [4].

Skin aging is generally categorized into two main types: intrinsic (chronological) aging and extrinsic (environmentally induced) aging. Intrinsic aging is a genetically programmed, inevitable process that occurs with age. It is associated with decreased proliferative capacity of dermal fibroblasts, diminished collagen production, reduced epidermal turnover, and lower levels of structural proteins such as elastin and laminin. In contrast, extrinsic aging is driven by chronic exposure to external factors, most notably UV radiation, which induces oxidative stress and inflammatory responses in skin cells. Among all extrinsic factors, UV-induced photoaging is recognized as the predominant cause of premature skin aging [5].

Upon UV exposure, skin cells release pro-inflammatory cytokines such as TNF-α, IL-6, and IL-8, which trigger the overproduction of ROS [6,7]. Excessive ROS generation overwhelms the skin’s endogenous antioxidant defense system and leads to cellular oxidative stress. This cascade not only damages cellular macromolecules such as lipids, proteins, and DNA but also activates signaling pathways, including the MAPKs and nuclear factor kappa B (NF-κB) pathways. Consequently, these processes upregulate matrix metalloproteinases (MMPs), particularly MMP-1, which degrades type I collagen, and downregulate collagen synthesis by dermal fibroblasts [8,9]. This imbalance results in ECM degradation and progressive weakening of dermal structure, ultimately contributing to the visible manifestations of photoaged skin.

As public awareness of skin health and cosmetic appearance increases, there is growing interest in discovering novel, safe, and effective agents for delaying or preventing skin aging. In particular, the focus has shifted toward natural products and plant-derived compounds that exhibit antioxidant, anti-inflammatory, and collagen-preserving activities. These compounds are gaining popularity not only due to their biological efficacy but also for their safety and environmental sustainability [10].

*C. japonicum* is a deciduous tree native to East Asia and the sole representative of the genus *Cercidiphyllum*. Traditionally known for its ornamental value, this species has more recently garnered scientific interest due to its phytochemical constituents, particularly flavonoids [11]. Flavonoids are a diverse group of polyphenolic compounds widely distributed in plants, recognized for their potent antioxidant, anti-inflammatory, anti-allergic, and anti-viral properties [12]. These compounds have been extensively studied for their protective roles in various disease models, including inflammation, cardiovascular disease, and cancer. However, despite the known health benefits of flavonoids, the pharmacological potential of those specifically derived from *C. japonicum*, especially in the context of skin aging, remains largely unexplored.

In our previous study, we successfully isolated and identified four major flavonoid-related compounds—maltol, chlorogenic acid, ellagic acid, and quercitrin—from the aerial parts of *Cercidiphyllum japonicum* using high-performance liquid chromatography (HPLC). These compounds exhibited notable antioxidant activity in vitro. Among them, quercitrin demonstrated a particularly strong effect in promoting cell proliferation in the E-screen assay, a standard method for evaluating estrogen-like activity, suggesting its potential utility in alleviating menopausal symptoms and enhancing skin regeneration capacity [13].

Each of these compounds has been reported to exert diverse biological functions beyond antioxidant effects. Maltol, a naturally occurring organic compound found in various plants including ginseng, possesses neuroprotective properties, such as inhibition of neuronal apoptosis and amelioration of memory impairment in models of age-related cognitive decline and neurodegenerative diseases [14,15]. Chlorogenic acid, a predominant polyphenol in coffee and several fruits, is well known for its anti-inflammatory, anti-obesity, hepatoprotective, and neuroprotective effects, in addition to its regulatory role in glucose and lipid metabolism, making it a promising candidate for metabolic syndrome management [16]. Ellagic acid, a polyphenolic compound widely distributed in berries and nuts, is recognized for its anticancer, anti-inflammatory, and neuroprotective activities. It modulates signaling pathways involved in cell proliferation and apoptosis and has shown therapeutic potential in neurodegenerative disease models such as Alzheimer’s and Parkinson’s disease [17,18,19]. Quercitrin, a glycosidic form of quercetin found in many medicinal plants, has been reported to exert anti-inflammatory, anti-allergic, and antimicrobial properties. It attenuates allergic dermatitis and suppresses the expression of pro-inflammatory cytokines in both in vitro and in vivo models, and additionally possesses tyrosinase-inhibiting and skin-lightening effects [20,21,22].

Building on these findings, the present study aims to investigate the protective effects of *C. japonicum*-derived compounds in human dermal fibroblasts subjected to TNF-α-induced inflammatory and oxidative stress conditions. Specifically, we assessed the ability of the selected compounds to attenuate oxidative stress, inhibit inflammatory signaling pathways, suppress MMP-1 expression, and enhance collagen synthesis. By elucidating the underlying mechanisms, this study seeks to evaluate the potential of *C. japonicum* as a natural source of anti-aging agents for skin health applications.

## 2. Materials and Methods

### 2.1. Plant Material

The aerial parts (stems and leaves) of *C. japonicum* were collected from July to September 2022. The plant materials were provided and characterized by Dr. C.H. Choi at the Gyeonggi-do Forestry Environment Research Center, Osan, Republic of Korea. The voucher specimens were deposited at the herbarium of the Gyeonggi-do Forestry Environment Research Center, Osan, Republic of Korea [13].

### 2.2. Apparatus, Reagents, and Chemicals

For reflux extraction, a multi-heating mantle (MS-EAM9023-03, Misung Scientific Co., Kyunggi-do, Republic of Korea) and a cooling water circulation device (CA-1116A, EYELA, Tokyo, Japan) were used. Extracts were prepared using a water bath (OSB-2200, EYELA) and rotary evaporator vacuum decompression extraction equipment (N-1100, EYELA). An oven temperature control system was purchased from WiseVen Won-155 (Daihan Scientific, Wonju, Republic of Korea). High-performance liquid chromatography (HPLC; Waters Alliance 2695 Separations Module, Milford, MA, USA) and an autosampler, photodiode array (PDA; Waters 2998 Photodiode Array Detector, Milford, MA, USA) were used for the quantitative analysis of FRs 4. HPLC (Agilent 1260 Infinity II Quat Pump, Santa Clara, CA, USA) and a variable-wavelength detector (Agilent Variable Wavelength Detector, Santa Clara, CA, USA) were used to obtain the results. A microplate spectrophotometer (Epoch, BioTek, Winooski, VT, USA) was used for biological activity analysis. Ethanol (EtOH) was purchased from Samchun Chemical Co. (Pyeongtaek, Republic of Korea), and the acetonitrile (ACN), methanol (MeOH), and water used for HPLC analysis were obtained from J. T. Baker. The positive control group used in the E-screen assay was Queens One, including dried red clover extract (Kyungjin Pharmaceutical Co., Ltd., Incheon, Republic of Korea), a commercially available drug used to improve menopausal symptoms. Compounds 1–4 (1, maltol; 2, chlorogenic acid; 3, ellagic acid; 4, quercitrin) were provided by the Natural Product Institute of Science and Technology (www.nist.re.kr accessed on 27 April 2024), Anseong, Republic of Korea [13].

### 2.3. Sample Extraction

Dried samples of *Cercidiphyllum japonicum*, weighing 10 or 23 g each, were collected and subjected to three extraction cycles lasting for three hours each. The extraction process was carried out in a reflux environment at temperatures ranging from 78 to 85 °C using EtOH as the extraction solvent. Following extraction, the solvent was filtered through filter paper (pore size: 0.45 μm), and the resulting solution was evaporated using a decompression concentrator at 55 °C to yield an EtOH extract [13].

### 2.4. Preparation of HPLC Samples and HPLC Condition

For quantitative analysis, 5 mg of *C. japonicum* extract was accurately weighed and dissolved in 1 mL of methanol (MeOH) to prepare a sample solution with a concentration of 5 mg/mL. The solution was subjected to ultrasonic treatment for 20 min to ensure complete dissolution, followed by filtration through a 0.45 μm PVDF membrane filter prior to injection.

HPLC analysis was performed using a reversed-phase INNO C18 column (250 × 4.6 mm, 5 μm particle size) at a column temperature of 30 °C. The injection volume was set to 10 μL. The mobile phase consisted of 0.1% trifluoroacetic acid in water (mobile phase A) and acetonitrile (mobile phase B), delivered at a flow rate of 1.0 mL/min. Detection was conducted at 254 nm. 

Gradient elution was programmed as follows: initial 95% mobile phase A for 10 min; linear decrease to 70% mobile phase A from 10 to 20 min; further gradient to 30% mobile phase A from 20 to 40 min; decrease to 0% mobile phase A from 40 to 42 min; hold at 100% mobile phase B until 44 min; return to 95% mobile phase A from 44 to 50 min; and maintained at 95% mobile phase A for an additional 10 min (Figure S1, Tables S1 and S2) [13].

### 2.5. Cell Viability

NHDFs were seeded in 96-well plates at a clear bottom (1 × 10^4^ cells/well) and incubated for 24 h. The isolated compounds were exposed to specified concentrations and incubated for 24 h in a cell incubator. After 24 h, the measurement of cell viability was conducted with EZ-Cytox solution. The absorbance was measured at 450 nm using a plate reader.

### 2.6. ROS Assay

NHDFs were seeded in black 96-well plates at a flat bottom (1 × 10^4^ cells/well) and incubated them for 24 h. The cells were stored in serum-free medium to create starvation conditions. After 24 h, the cells were treated with the samples for 1 h and then exposed to 20 ng/mL TNF-α for 15 min. The cell was stained with 2′,7′-dichlorodihydrofluorescein diacetate (DCFDA) for 15 min. Subsequently, the treated NHDFs were washed with PBS after removing the supernatant. The measurement of fluorescence was conducted using a plate reader at 485 nm and 530 nm.

### 2.7. Enzyme-Linked Immunosorbent Assay (ELISA)

NHDFs were seeded in 48-well plates at a flat bottom (2 × 10^4^ cells/well) and were incubated for 24 h. The cells were stored in serum-free medium to create starvation conditions. After 24 h, the cells were treated with the samples for 1 h and then exposed to 20 ng/mL TNF-α for 24 h. Secretion of MMP-1 and pro-collagenα1 was measured using ELISA kits. The optical density was measured using a microplate reader at 450 nm.

### 2.8. Western Blotting

NHDFs were seeded in 6-well plates at a flat bottom (3 × 10^5^ cells/well) and were incubated for 24 h. To induce starvation conditions, the cells were stored in serum-free medium for 24 h. Then, the cells were exposed to Ellagic acid for 1 h and treated with 20 ng/mL TNF-α. The cells were harvested after 15 min to evaluate the levels of phospho-ERK, ERK, phospho-p38, p38, phospho-JNK, JNK, and GAPDH. Additionally, cells were collected 6 h after treatment with TNF-α to assess the level of COX-2, HO-1, and GAPDH. The cells were lysed using 1X Radioimmunoprecipitation Analysis (RIPA) buffer (Tech & Innovation, Gangwon, Republic of Korea), and the soluble fractions were used as protein samples. Protein concentrations were determined using a bicinchoninic acid (BCA) protein assay kit (Merck). Protein levels were analyzed by Western blotting using primary antibodies against phospho-ERK, ERK, phospho-p38, p38, phospho-JNK, JNK, HO-1, COX-2, and GAPDH (Cell Signaling Technology, Danvers, MA, USA) which were allowed to react for 4 h, at room temperature. Secondary antibodies, goat anti-rabbit IgG-HRP (Cell Signaling Technology, Danvers, MA, USA), were incubated at room temperature for 1 h. Protein bands were visualized using the SuperSignal West Femto Maximum Sensitivity Chemiluminescent Substrate (Thermo Fisher Scientific, Waltham, MA, USA) and the Fusion Solo Chemiluminescent System (PEQLAB Biotechnologie GmbH, Erlangen, Germany) [23].

### 2.9. Real-Time PCR

NHDFs were seeded in 6-well plates at a flat bottom (3 × 10^5^ cells/well) and were incubated for 24 h. The medium was then replaced with a serum-free medium to starve the cells for 24 h. The cells were exposed to Ellagic acid for 1 h and treated with 20 ng/mL TNF-α. The cells were collected after 4 h to evaluate the levels of IL-6, IL-8, and β-actin. Then, cellular RNA was isolated using a RNeasy Mini Kit (QIAGEN, Hilden, Germany). Complementary DNA (cDNA) was synthesized using a RevertAid First Strand cDNA synthesis Kit (Thermoscientific, Waltham, MA, USA). Real-time PCR was performed using a TOPreal^TM^ SYBR Green qPCR High-ROX Premix (Enzynomics, Daejeon, Republic of Korea) [24].

### 2.10. Statistical Analysis

Data are presented as mean ± standard error of the means (SEMs). Statistical significance was assessed using a one-way analysis of variance (ANOVA) conducted with GraphPad Prism version 5. Tukey’s multiple-comparison test was applied to evaluate differences between groups, with statistical significance set at *p* < 0.05.

## 3. Results

### 3.1. Effects of Compounds of C. japonicum on Viability of NHDFs

**Figure 1:**
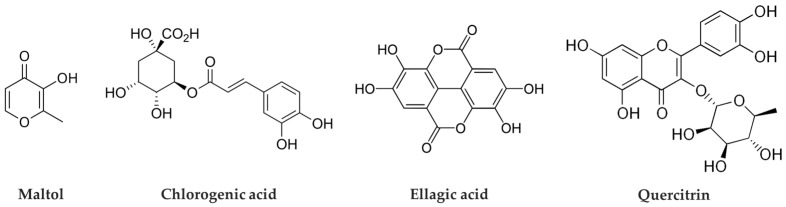
Chemical structures of phytochemicals identified in *Cercidiphyllum japonicum*: maltol, chlorogenic acid, ellagic acid, and quercitrin.

Phytochemical analysis of *C. japonicum* identified four major compounds: maltol, chlorogenic acid, ellagic acid, and quercitrin (Figure 1). Quantitative HPLC analysis was performed to determine the content of these compounds in the extract. Among them, chlorogenic acid was the most abundant, present at 17.83 ± 0.65 mg/g, followed by ellagic acid at 5.03 ± 0.02 mg/g, maltol at 4.68 ± 0.03 mg/g, and quercitrin at 1.06 ± 0.00 mg/g. All four compounds exhibited strong antioxidant activity; notably, quercitrin showed excellent cell proliferation-promoting effects in the E-screen assay. These findings suggest that quercitrin may serve as a key indicator compound for the potential alleviation of menopausal symptoms [10]. Furthermore, based on the strong antioxidant activity of *C. japonicum*, subsequent studies were conducted to investigate its potential effects on skin antioxidant capacity and anti-aging properties.

**Figure 2:**
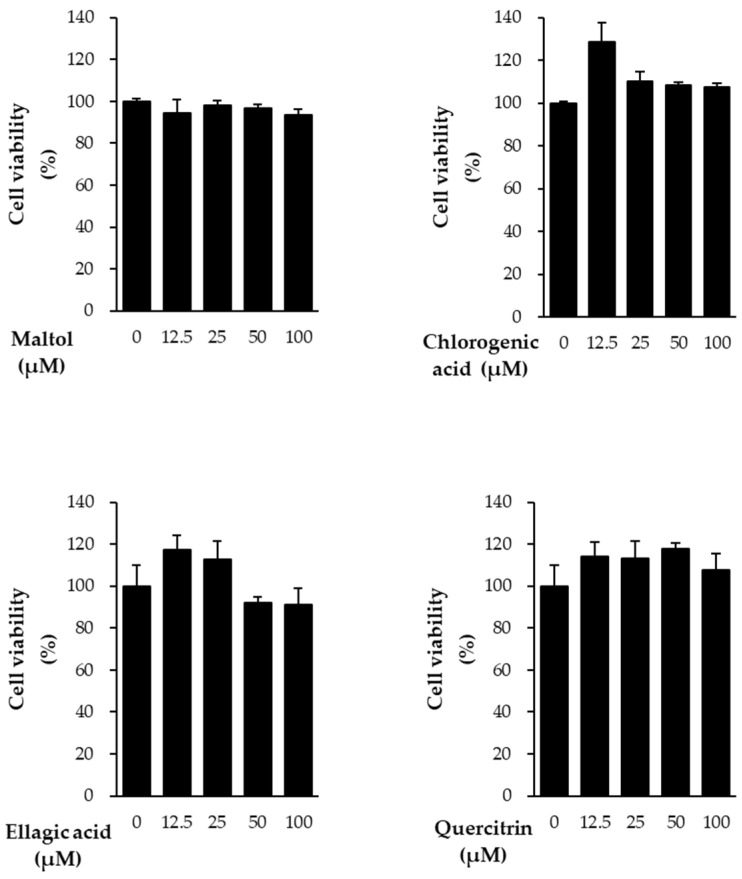
Cell viability of NHDFs following treatment with various concentrations of the compounds. NHDFs were seeded at a density of 1 × 10^4^ cells/well in 96-well plates and incubated for 24 h. Cells were then treated with the indicated concentrations of each compound for an additional 24 h. Cell viability was assessed using EZ-Cytox reagent, and the results are expressed as a percentage relative to the vehicle control. Data are presented as mean ± SEM from at least three independent experiments.

In this study, we evaluated the protective effects of the compounds against TNF-α-induced damage in NHDFs. Prior to assessing these effects, cell viability assays were conducted to determine the cytotoxicity of the compounds. All four compounds showed no cytotoxicity at concentrations below 100 μM (Figure 2). Based on these results, subsequent experiments were performed using a concentration of 10 μM.

### 3.2. Effects of Compounds on Intracellular ROS Secretion in NHDFs

The effects of the compounds on intracellular ROS generation were evaluated in NHDFs following stimulation with TNF-α. Cells treated with 20 ng/mL TNF-α for 24 h exhibited a significant increase in ROS production, as evidenced by a 2.26 ± 0.08-fold rise in fluorescence intensity compared to the untreated control group (*p* < 0.001). This result confirms that TNF-α effectively induces oxidative stress in dermal fibroblasts.

**Figure 3:**
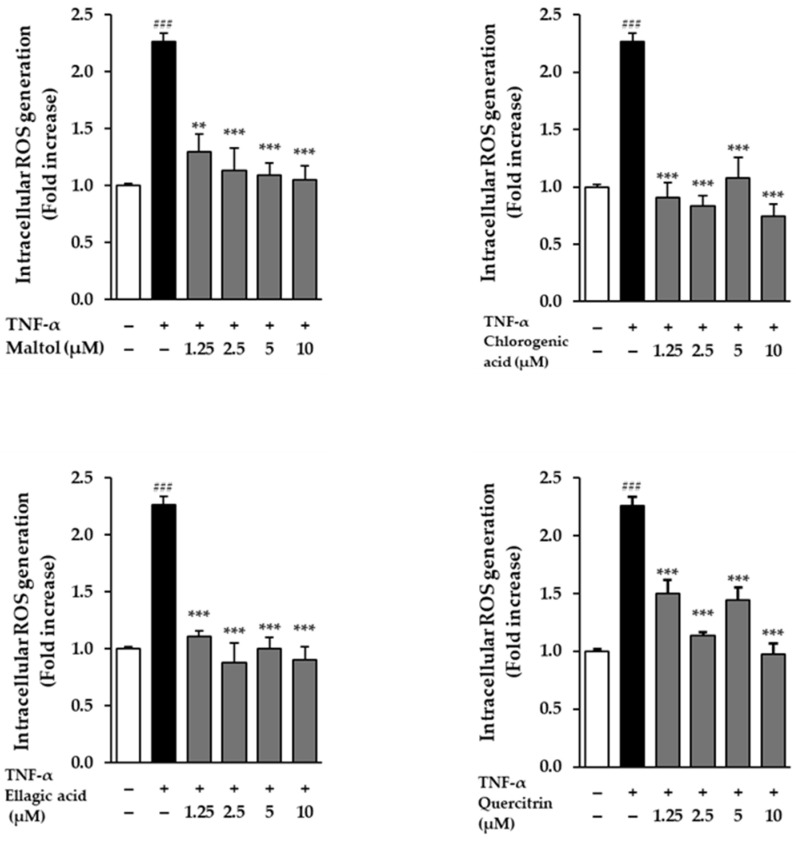
Effects of compounds on intracellular ROS accumulation in TNF-α-stimulated NHDFs. NHDFs were seeded at a density of 1 × 10^4^ cells/well in 96-well black plates and incubated for 24 h. The medium was then replaced with serum-free medium for an additional 24 h to induce starvation. Cells were pretreated with the compounds for 1 h, followed by stimulation with 20 ng/mL TNF-α for 15 min. Subsequently, cells were stained with dichlorofluorescein diacetate (DCFDA) for 15 min. Fluorescence intensity, indicating intracellular ROS levels, was measured using an EnSpire Multimode Plate Reader and expressed as a percentage relative to the vehicle control. Data are presented as mean ± SEM from three independent experiments. ^###^ *p* < 0.001 vs. vehicle control; ** *p* < 0.01, *** *p* < 0.001 vs. TNF-α-treated control.

To assess the antioxidant potential of the test compounds, NHDFs were co-treated with TNF-α and each compound. All four compounds demonstrated a reduction in TNF-α-induced ROS levels, indicating their capacity to attenuate oxidative stress. Among them, ellagic acid showed a particularly pronounced effect, significantly suppressing ROS production in a dose-dependent manner. Fluorescence microscopy and quantitative analysis revealed a gradual decline in fluorescence intensity with increasing concentrations of ellagic acid, supporting its strong ROS-scavenging activity (Figure 3). These findings suggest that the compounds, especially ellagic acid, may protect dermal fibroblasts from TNF-α-induced oxidative damage through their antioxidant properties.

### 3.3. Effects of Compounds on MMP-1 and Pro-Collagen Type Ι α1 Protein Secretion in TNF-α-Treated NHDFs

**Figure 4:**
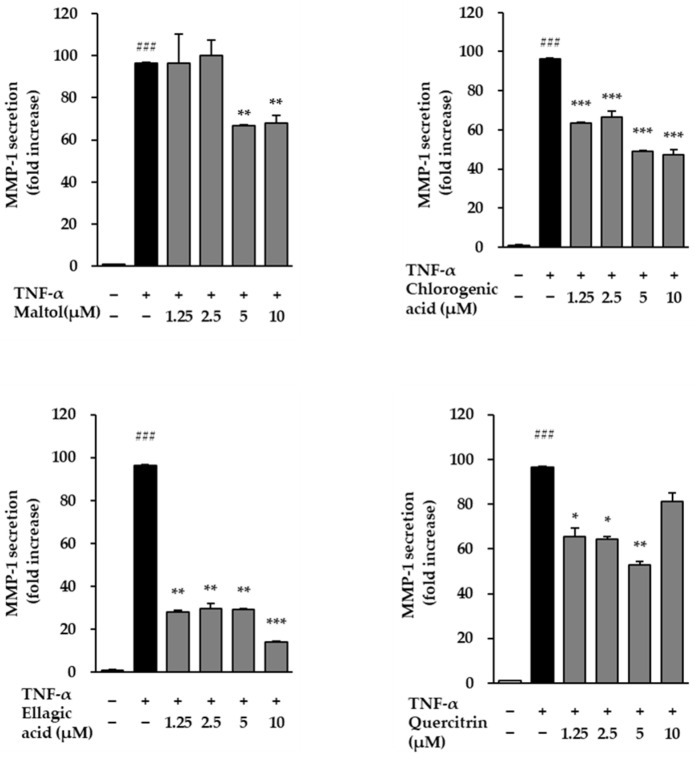
Effects of compounds on MMP-1 secretion in TNF-α-treated NHDFs. NHDFs were seeded at a density of 2 × 10^4^ cells/well in 48-well plates and incubated for 24 h. The medium was then replaced with serum-free medium for 24 h to induce starvation. Cells were pretreated with the compounds for 1 h, followed by stimulation with 20 ng/mL TNF-α for 24 h. MMP-1 levels in the culture supernatants were quantified using an ELISA kit and are presented as fold increase relative to the TNF-α-treated group. Data are expressed as mean ± SEM from two independent experiments. ^###^ *p* < 0.001 vs. vehicle control; * *p* < 0.05, ** *p* < 0.01, *** *p* < 0.001 vs. TNF-α-treated control.

We evaluated the effects of the compounds on the secretion levels of MMP-1 and Pro-collagen Type I α1 in NHDFs following TNF-α stimulation. Treatment with TNF-α (20 ng/mL) for 24 h significantly increased MMP-1 secretion compared to the untreated control group, reaching 96.71 ± 0.36 ng/mL (*p* < 0.001) (Figure 4). This result indicates that TNF-α induces ECM degradation by upregulating MMP-1, a key enzyme responsible for collagen breakdown in skin aging and inflammatory conditions.

To determine the protective effects of the test compounds, cells were co-treated with TNF-α and individual compounds. All four compounds markedly reduced MMP-1 secretion compared to the TNF-α-treated group, suggesting that these compounds may prevent ECM degradation by inhibiting MMP-1 expression.

**Figure 5:**
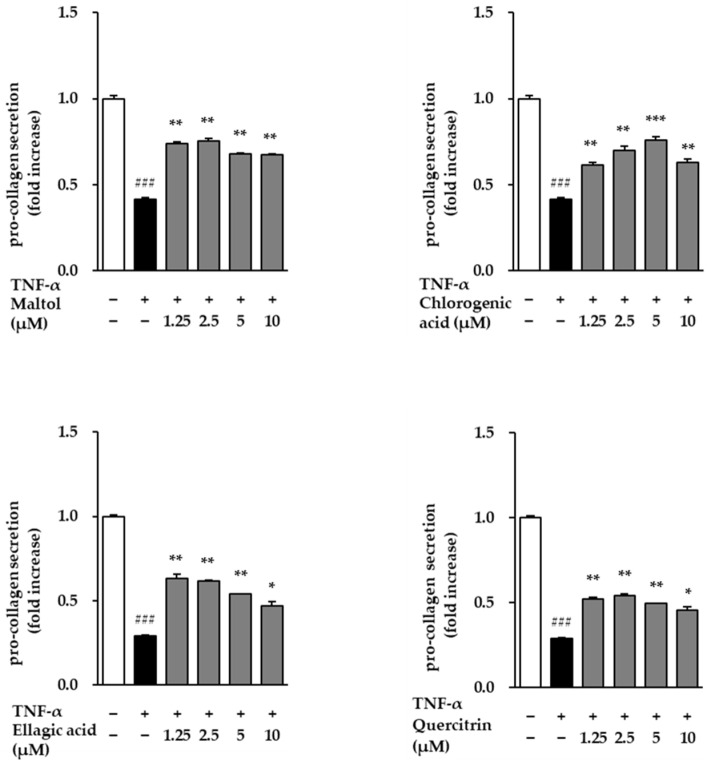
Effects of compounds on type I procollagen secretion in TNF-α-treated NHDFs. NHDFs were seeded at a density of 2 × 10^4^ cells/well in 48-well plates and incubated for 24 h. The medium was then replaced with serum-free medium for 24 h to induce starvation. Cells were pretreated with the compounds for 1 h, followed by stimulation with 20 ng/mL TNF-α for 24 h. Type I procollagen levels in the culture supernatants were quantified using an ELISA kit and are presented as fold increase relative to the TNF-α-treated group. Data are expressed as mean ± SEM from two independent experiments. ^###^ *p* < 0.001 vs. vehicle control; * *p* < 0.05, ** *p* < 0.01, *** *p* < 0.001 vs. TNF-α-treated control.

Conversely, Pro-collagen Type I α1 secretion—a marker of collagen synthesis—was significantly suppressed in the TNF-α-treated group, with levels decreasing to 0.29 ± 0.01 ng/mL (*p* < 0.001) relative to the control (Figure 5). However, treatment with the test compounds attenuated this TNF-α-induced reduction and led to a concentration-dependent restoration of Pro-collagen Type I α1 secretion. These results suggest that the compounds not only inhibit TNF-α-induced collagen degradation but also promote collagen synthesis, indicating their potential protective effects against inflammation-induced skin aging.

### 3.4. Spider Chart for Evaluating the Efficacy of the Compounds in TNF-α-Treated NHDFs

**Figure 6:**
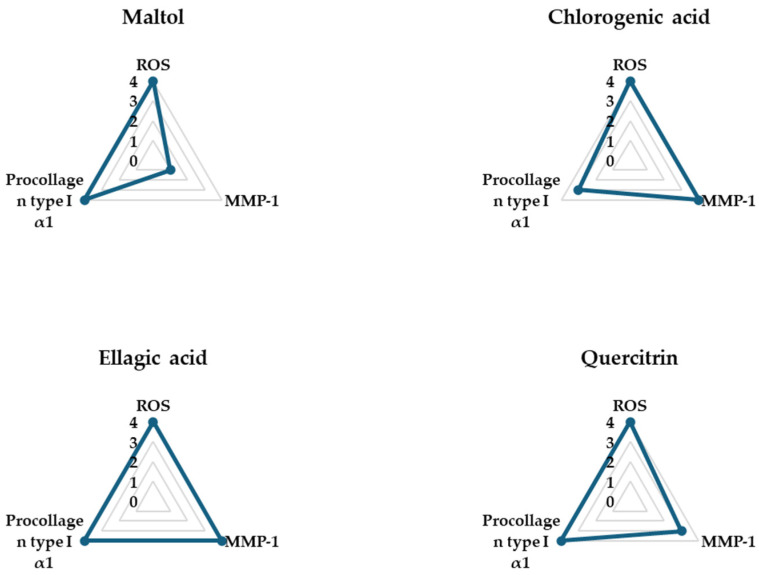
Spider chart comparing the anti-aging efficacy of the tested compounds in TNF-α-stimulated NHDFs. The chart integrates three key biological parameters: intracellular ROS levels, MMP-1 secretion, and pro-collagen type I α1 production. Values were normalized and plotted to visually compare the overall protective effects of each compound against TNF-α-induced skin aging.

The spider charts in Figure 6 provide a comprehensive visual comparison of the effects of four phytochemical compounds—maltol, chlorogenic acid, ellagic acid, and quercitrin—on three key biomarkers involved in TNF-α-induced skin aging in NHDFs: intracellular ROS, pro-collagen type I α1, and MMP-1. These biomarkers were selected as representative indicators of oxidative stress, collagen synthesis, and collagen degradation, respectively.

Each axis of the spider chart represents a scaled response for a single biomarker, where higher ROS and MMP-1 levels reflect pro-aging effects, while higher levels of pro-collagen type I α1 indicate anti-aging or skin-repair potential. The plotted values were normalized to allow for direct comparison across compounds.

Among the four compounds, ellagic acid showed the most favorable profile, achieving the maximum score (4) in all three parameters—demonstrating strong ROS suppression, significant inhibition of MMP-1 secretion, and notable upregulation of pro-collagen type I α1 secretion. These results suggest that these three compounds exhibit comprehensive protective effects against TNF-α-induced skin damage, by not only mitigating oxidative stress and collagen breakdown but also promoting collagen production.

Additionally, as no prior studies have specifically evaluated the antioxidant and anti-inflammatory effects of ellagic acid in TNF-α-stimulated NHDFs, we selected ellagic acid for subsequent mechanistic investigations to further explore its potential protective effects.

### 3.5. Effects of Ellagic Acid on Intracellular ROS Secretion in NHDFs

**Figure 7:**
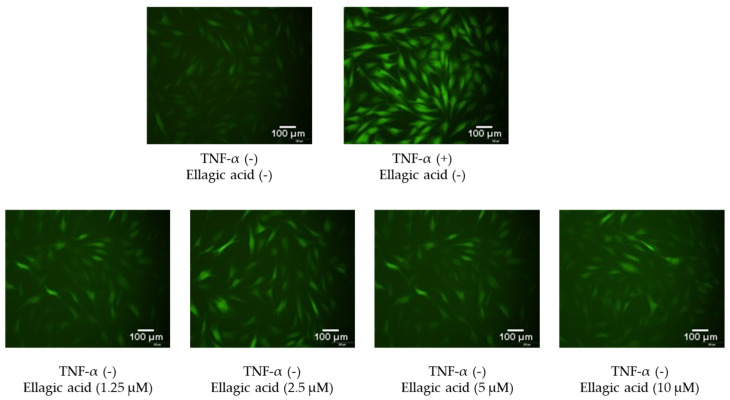
Inhibitory effects of ellagic acid on TNF-α-induced intracellular ROS accumulation in NHDFs. Cells were stained with dichlorofluorescein diacetate (DCFDA) to visualize reactive oxygen species, and fluorescence images were captured using a fluorescence microscope.

Figure 7 shows the effects of ellagic acid on intracellular ROS accumulation in TNF-α-stimulated NHDFs, visualized using DCFDA staining and fluorescence microscopy. In the control group, cells exhibited baseline levels of green fluorescence, indicating low basal ROS levels. Upon TNF-α treatment, a marked increase in fluorescence intensity was observed, reflecting a significant elevation in intracellular ROS production due to inflammatory stress.

However, co-treatment with ellagic acid led to a noticeable reduction in fluorescence intensity compared to the TNF-α group alone. This decrease was evident across all compound-treated groups, suggesting that ellagic acid effectively suppressed TNF-α-induced ROS generation in a dose-dependent manner. These findings support the potent antioxidant activity of ellagic acid in protecting dermal fibroblasts from oxidative stress.

### 3.6. Effects of Ellagic Acid on MAPK Phosphorylation in TNF-α-Treated NHDFs

**Figure 8:**
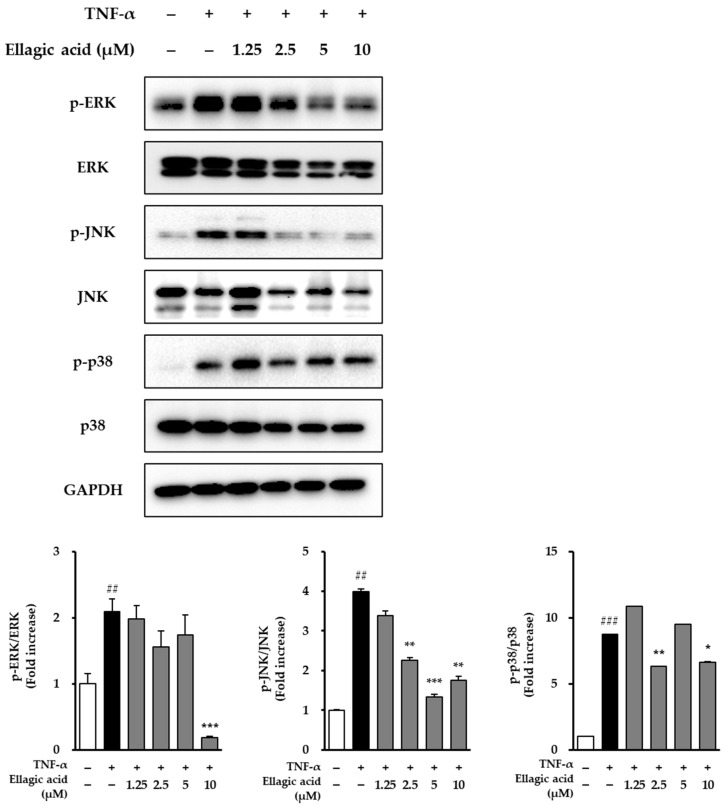
Effects of ellagic acid on TNF-α-induced phosphorylation of MAPKs in NHDFs. Cells were pretreated with ellagic acid at concentrations of 1.25, 2.5, 5, or 10 μM for 1 h, followed by stimulation with 20 ng/mL TNF-α for 15 min. Protein levels of phospho-JNK, JNK, phospho-ERK, ERK, phospho-p38, p38, and GAPDH were analyzed by immunoblotting. The phosphorylation levels of MAPKs are represented as fold changes compared to the control. Data are presented as mean ± SEM (n = 2). ^##^ *p* < 0.01, ^###^ *p* < 0.001 vs. vehicle control; * *p* < 0.05, ** *p* < 0.01, *** *p* < 0.001 vs. TNF-α-treated group.

As shown in Figure 8, the effects of ellagic acid on the phosphorylation of MAPKs were evaluated in NHDFs stimulated with TNF-α. Upon treatment with 20 ng/mL TNF-α for 30 min, phosphorylation levels of all three major MAPK pathways—JNK, ERK, and p38—were markedly elevated compared to the untreated control group. Specifically, ERK phosphorylation increased by 2.09 ± 0.19-fold (*p* < 0.01), JNK phosphorylation by 3.99 ± 0.06-fold (*p* < 0.01), and p38 phosphorylation by 8.72 ± 0.03-fold (*p* < 0.001), indicating that TNF-α strongly activates MAPK signaling associated with inflammation and cellular stress responses.

To assess the inhibitory effect of ellagic acid on this activation, NHDFs were co-treated with ellagic acid at varying concentrations along with TNF-α. Ellagic acid treatment resulted in a dose-dependent suppression of JNK phosphorylation, suggesting that it interferes with TNF-α-mediated stress signaling. While detailed quantification of ERK and p38 suppression was not included, the observed trend indicates that ellagic acid may attenuate MAPK pathway activation, particularly by targeting JNK, a key mediator in inflammation-induced skin damage and aging. These findings suggest that ellagic acid exerts anti-inflammatory and potential anti-aging effects in dermal fibroblasts, at least in part, through modulation of MAPK signaling pathways.

### 3.7. Effects of Ellagic Acid on COX-2 and HO-1 in TNF-α-Treated NHDFs

**Figure 9:**
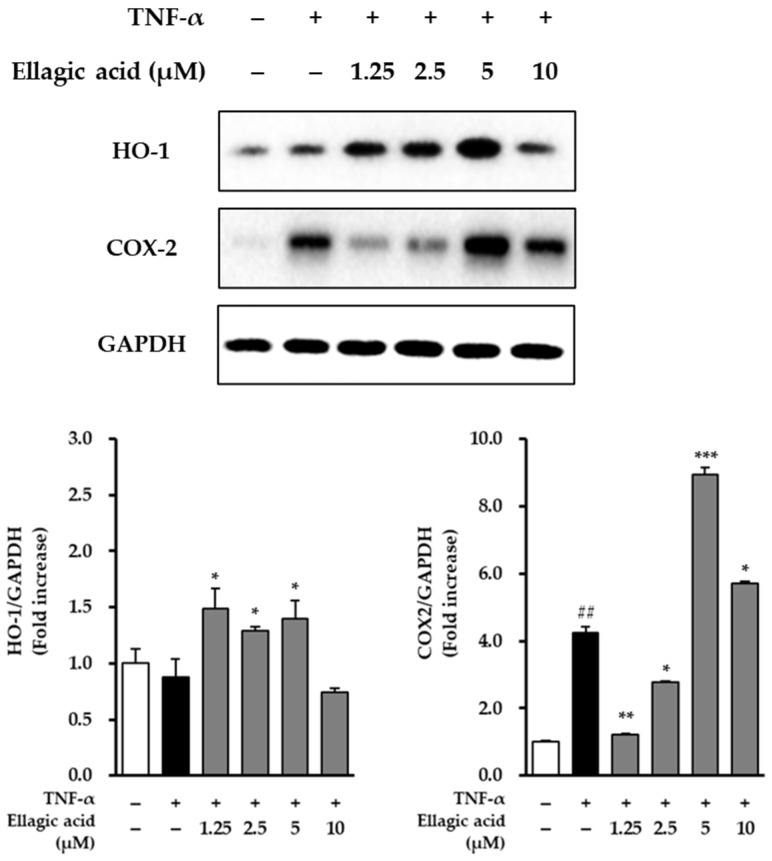
Effects of ellagic acid on the expression of COX-2 and HO-1 in TNF-α-treated NHDFs. Cells were pretreated with ellagic acid at concentrations of 1.25, 2.5, 5, or 10 μM for 1 h, followed by stimulation with 20 ng/mL TNF-α for 6 h. Protein expression levels of COX-2 and HO-1 were analyzed by immunoblotting, with GAPDH used as a loading control. Densitometric analysis results are expressed as fold change relative to the control. Data are presented as mean ± SEM (*n* = 2). ^##^ *p* < 0.05 vs. vehicle control; * *p* < 0.05,** *p* < 0.01, *** *p* < 0.001 vs. TNF-α-treated group.

As shown in Figure 9, the modulatory effects of ellagic acid on the expression of COX-2 and HO-1 were examined in TNF-α-stimulated NHDFs. Treatment with TNF-α (20 ng/mL) significantly upregulated the expression of COX-2, showing a 4.24 ± 0.18-fold increase compared to the untreated control group (*p* < 0.01), indicating a strong pro-inflammatory response. Conversely, TNF-α treatment led to a reduction in HO-1 expression, decreasing to 0.88 ± 0.11-fold relative to the control, suggesting that TNF-α may suppress cytoprotective antioxidant responses.

Upon co-treatment with ellagic acid, COX-2 expression was significantly suppressed. At a concentration of 1.25 μM, ellagic acid reduced COX-2 expression to 1.20 ± 0.05-fold compared to the TNF-α-only group (*p* < 0.01), demonstrating its anti-inflammatory potential. In parallel, ellagic acid treatment restored and enhanced HO-1 expression in a concentration-dependent manner, indicating the compound’s role in promoting antioxidant defense mechanisms.

These results suggest that ellagic acid exerts dual regulatory effects in TNF-α-stimulated dermal fibroblasts by downregulating pro-inflammatory COX-2 and upregulating cytoprotective HO-1, highlighting its potential as a therapeutic agent against inflammation-induced skin damage.

### 3.8. Effect of Ellagic Acid on Pro-Inflammatory Cytokines in TNF-α-Treated NHDFs

The effects of ellagic acid on the mRNA expression levels of pro-inflammatory cytokines were evaluated in TNF-α-stimulated NHDFs. TNF-α treatment significantly increased the expression of IL-6 and IL-8 mRNA compared to the untreated control group. Specifically, IL-6 expression increased by 2.65 ± 0.07-fold (*p* < 0.05), while IL-8 expression showed a marked elevation of 52.83 ± 3.78-fold (*p* < 0.01), indicating a robust inflammatory response.

**Figure 10:**
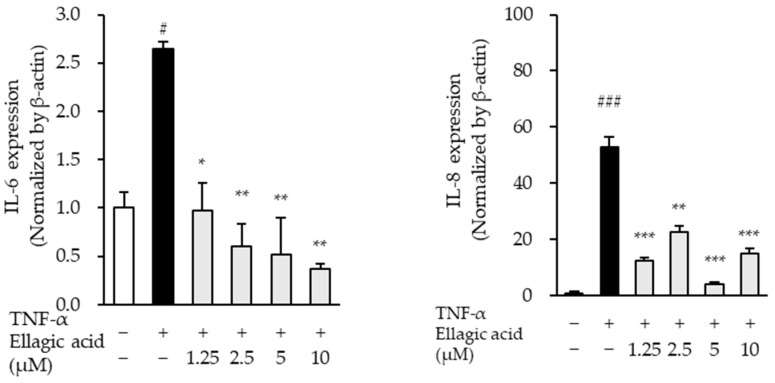
Effects of ellagic acid on the mRNA expression of IL-6 and IL-8 in TNF-α-treated NHDFs. Cells were pretreated with ellagic acid at concentrations of 1.25, 2.5, 5, or 10 μM for 1 h, followed by stimulation with 20 ng/mL TNF-α for 4 or 24 h. mRNA levels were quantified and expressed as fold change relative to the untreated control. Data are presented as mean ± SEM (n = 3). ^#^ *p* < 0.05, ^###^ *p* < 0.001 vs. vehicle control; * *p* < 0.05, ** *p* < 0.01, *** *p* < 0.001 vs. TNF-α-treated group.

Co-treatment with ellagic acid led to a concentration-dependent downregulation of IL-6, and IL-8 mRNA expression (Figure 10). Notably, IL-8 expression was most significantly suppressed across all tested concentrations, suggesting that ellagic acid may exert potent anti-inflammatory effects, particularly by targeting IL-8, a key chemokine involved in neutrophil recruitment and inflammation amplification. These results support the role of ellagic acid in modulating inflammatory cytokine expression in TNF-α-induced skin inflammation models.

## 4. Discussion

As concerns about skin aging continue to grow, there is increasing interest in utilizing natural bioactive compounds for anti-aging applications. In this study, we investigated the protective effects of flavonoid compounds derived from *Cercidiphyllum japonicum* in TNF-α-stimulated normal human dermal fibroblasts (NHDFs), with a particular focus on their antioxidant and anti-inflammatory potential.

Among the four major constituents analyzed—maltol, chlorogenic acid, ellagic acid, and quercitrin—all significantly reduced intracellular reactive oxygen species (ROS) levels in TNF-α-treated cells, indicating strong antioxidant activity and the ability to alleviate oxidative stress, a key contributor to skin aging [25,26]. Beyond their antioxidant properties, these compounds also modulated extracellular matrix (ECM) remodeling by decreasing matrix metalloproteinase-1 (MMP-1) secretion and increasing pro-collagen type I α1 levels [27].

While all four compounds exhibited comparable antioxidant effects, ellagic acid was selected for further mechanistic studies due to its superior dual effect on MMP-1 inhibition and collagen I stimulation in preliminary analyses. Although ellagic acid has been widely recognized for its antioxidative capacity, its specific role in regulating dermal fibroblast responses under inflammatory conditions has not been thoroughly investigated [28]. Therefore, we conducted additional experiments to elucidate the underlying molecular mechanisms of ellagic acid in TNF-α-stimulated NHDFs.

Ellagic acid effectively inhibited the phosphorylation of key mitogen-activated protein kinases (MAPKs)—JNK, ERK, and p38—which are typically activated in response to inflammatory and oxidative stress. Given that MAPK activation leads to increased transcriptional activity of AP-1 and NF-κB, which in turn promote MMP-1 expression and pro-inflammatory cytokine production, the suppression of this pathway by ellagic acid suggests a protective mechanism against extracellular matrix (ECM) degradation and skin inflammation [29,30]. These results support the hypothesis that ellagic acid mitigates TNF-α-induced skin aging by blocking MAPK-mediated signaling cascades.

We further investigated the expression of cyclooxygenase-2 (COX-2) and heme oxygenase-1 (HO-1), which serve as representative markers of inflammation and antioxidant defense, respectively. TNF-α stimulation significantly increased COX-2 expression while reducing HO-1 levels, consistent with a pro-inflammatory cellular state. Treatment with ellagic acid resulted in a dose-dependent downregulation of COX-2 and restoration of HO-1 expression, indicating its dual role in suppressing inflammatory signaling and enhancing cytoprotective antioxidant responses [31,32].

Additionally, ellagic acid significantly downregulated the mRNA expression of pro-inflammatory cytokines, including interleukin-6 (IL-6), interleukin-8 (IL-8), and interleukin-1β (IL-1β), with IL-8 showing the most pronounced decrease across all tested concentrations. These findings further confirm the anti-inflammatory efficacy of ellagic acid in TNF-α-stimulated dermal fibroblasts [33]. However, at higher concentrations (e.g., 5 μM), a slight increase in COX-2 and IL-1β expression was observed, suggesting a potential biphasic or concentration-dependent response. This observation underscores the need for further investigation to establish an optimal therapeutic range and avoid possible pro-inflammatory effects at higher doses [34].

**Figure 11:**
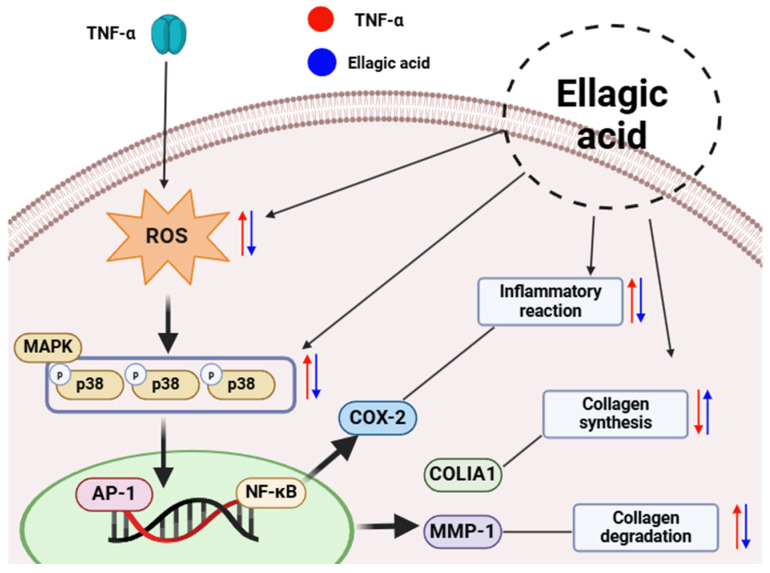
Schematic illustration of the protective effects of ellagic acid in TNF-α-stimulated NHDFs.

In summary, ellagic acid—a flavonoid component isolated from *Cercidiphyllum japonicum*—demonstrates potent antioxidant and anti-inflammatory properties in TNF-α-stimulated human dermal fibroblasts (Figure 11). It regulates multiple skin aging-related pathways, including oxidative stress, MAPK signaling, ECM remodeling, and cytokine production [35]. Although ellagic acid has limited systemic bioavailability due to its poor solubility and membrane permeability [36], recent studies have shown that it achieves effective transdermal delivery when formulated as a gel or complex, supporting its potential as a topical anti-aging agent [37]. These findings suggest that ellagic acid is a promising natural ingredient for skincare applications. Nevertheless, further mechanistic and in vivo validation studies are required to confirm its efficacy and safety in broader dermatological contexts [38].

## 5. Conclusions

This study demonstrated the antioxidant and anti-aging effects of four flavonoid compounds—maltol, chlorogenic acid, ellagic acid, and quercitrin—isolated from *Cercidiphyllum japonicum* in TNF-α-stimulated human dermal fibroblasts. All compounds significantly attenuated intracellular ROS levels, reduced MMP-1 secretion, and enhanced type I procollagen production, indicating their potential as protective agents against oxidative stress and extracellular matrix degradation. Among them, ellagic acid showed the most pronounced biological activity and was therefore selected for further mechanistic analysis.

Mechanistic investigations revealed that ellagic acid suppressed MAPK phosphorylation and COX-2 expression while upregulating HO-1, suggesting its ability to modulate key redox-sensitive and inflammatory signaling pathways implicated in skin aging. However, a slight pro-inflammatory response at higher concentrations highlights the need for further dose optimization and toxicity evaluation.

Although this study primarily focused on ellagic acid due to its superior efficacy, the observed activities of maltol, chlorogenic acid, and quercitrin suggest that these compounds also possess significant bioactive potential. Future studies should explore the synergistic or combinatorial effects of these flavonoids, which may enhance therapeutic efficacy in multi-component skin care formulations.

Several limitations should be acknowledged. The current study was conducted exclusively in vitro using a single cell type, which does not fully capture the complexity of skin architecture or in vivo biological interactions. In addition, the individual application of each flavonoid did not account for potential interactions among compounds. Furthermore, the pharmacokinetics, long-term safety, and dermal absorption profiles of ellagic acid remain to be elucidated.

In conclusion, ellagic acid represents a promising natural compound for incorporation into anti-aging skin care strategies. Nonetheless, its low solubility and limited bioavailability present challenges for topical application. Advanced formulation technologies, such as nanoencapsulation or complexation, may improve its dermal penetration and stability. Further in vivo studies and clinical trials are essential to confirm the efficacy, optimal dosage, and safety profile of ellagic acid, thereby facilitating its translation into practical and effective cosmetic applications.

## Data Availability

This published article contains all the data created or analyzed during this study. Any additional data or information can be made available to the corresponding author upon request.

## Supplementary Materials

The following supporting information can be downloaded at: https://www.mdpi.com/article/10.3390/cimb47080631/s1.

## Abbreviations

The following abbreviations are used in this manuscript:

*C. japonicum*	*Cercidiphyllum japonicum*
COX-2	Cyclooxygenase-2
DCFDA	Dichlorofluorescein Diacetate
ECM	Extracellular Matrix
ELISA	High-Performance Liquid Chromatography
HO-1	Heme Oxygenase-1
IL-6	Interleukin-6
IL-8	Interleukin-8
MAPKs	Mitogen-Activated Protein Kinases
MMP-1	Matrix Metalloproteinase-1
NHDF	Normal Human Dermal Fibroblast
ROS	Reactive Oxygen Species
TNF-α	Tumor Necrosis Factor-alpha
UV	Ultraviolet

## References

[1] Venus M., Waterman J., McNab I. (2010). Basic physiology of the skin. Surgery.

[2] Woo W.M. (2019). Skin structure and biology. Imaging Technol. Transdermal Deliv. Ski. Disord..

[3] Proksch E., Brandner J.M., Jensen J.M. (2008). The skin: An indispensable barrier. Exp. Dermatol..

[4] Cole M.A., Quan T., Voorhees J.J., Fisher G.J. (2018). Extracellular matrix regulation of fibroblast function: Redefining our perspective on skin aging. J. Cell Commun. Signal..

[5] Tobin D.J. (2017). Introduction to skin aging. J. Tissue Viability.

[6] Ciążyńska M., Olejniczak-Staruch I., Sobolewska-Sztychny D., Narbutt J., Skibińska M., Lesiak A. (2021). Ultraviolet radiation and chronic inflammation—Molecules and mechanisms involved in skin carcinogenesis: A narrative review. Life.

[7] Kim S.B., Kim J.E., Kang O.H., Mun S.H., Seo Y.S., Kang D.H., Yang D.W., Ryu S.Y., Lee Y.M., Kwon D.Y. (2015). Protective effect of ixerisoside A against UVB-induced pro-inflammatory cytokine production in human keratinocytes. Int. J. Mol. Med..

[8] Masaki H. (2010). Role of antioxidants in the skin: Anti-aging effects. J. Dermatol. Sci..

[9] Phung H.M., Lee S., Hong S., Lee S., Jung K., Kang K.S. (2021). Protective effect of polymethoxyflavones isolated from Kaempferia parviflora against TNF-α-induced human dermal fibroblast damage. Antioxidants.

[10] Ahmed I.A., Mikail M.A., Zamakshshari N., Abdullah A.-S.H. (2020). Natural anti-aging skincare: Role and potential. Biogerontology.

[11] Lee T.-S., Kim Y.-G., Lee H.-H., Kim Y.-S. (2015). Phenolic glycosides from *Cercidiphyllum japonicum* leaves. J. Korean Wood Sci. Technol..

[12] Pietta P.-G. (2000). Flavonoids as antioxidants. J. Nat. Prod..

[13] Cho H., Tran G.H., Ann H.W., Lee H.-D., Choi C.H., Lee S., Lee S. (2024). Estrogen-like cell proliferation abilities of Korea forest plant resources on MCF-7 cells and analysis of active compounds. Forests.

[14] Han Y., Xu Q., Hu J.-n., Han X.-y., Li W., Zhao L.-c. (2015). Maltol, a food flavoring agent, attenuates acute alcohol-induced oxidative damage in mice. Nutrients.

[15] Sha J.-y., Zhou Y.-d., Yang J.-y., Leng J., Li J.-h., Hu J.-n., Liu W., Jiang S., Wang Y.-p., Chen C. (2019). Maltol (3-hydroxy-2-methyl-4-pyrone) slows d-galactose-induced brain aging process by damping the Nrf2/HO-1-mediated oxidative stress in mice. J. Agric. Food Chem..

[16] Naveed M., Hejazi V., Abbas M., Kamboh A.A., Khan G.J., Shumzaid M., Ahmad F., Babazadeh D., FangFang X., Modarresi-Ghazani F. (2018). Chlorogenic acid (CGA): A pharmacological review and call for further research. Biomed. Pharmacother..

[17] Vattem D., Shetty K. (2005). Biological functionality of ellagic acid: A review. J. Food Biochem..

[18] Farbood Y., Sarkaki A., Dolatshahi M., Mansouri S.M.T., Khodadadi A. (2015). Ellagic acid protects the brain against 6-hydroxydopamine induced neuroinflammation in a rat model of Parkinson’s disease. Basic Clin. Neurosci..

[19] Jha A.B., Panchal S.S., Shah A. (2018). Ellagic acid: Insights into its neuroprotective and cognitive enhancement effects in sporadic Alzheimer’s disease. Pharmacol. Biochem. Behav..

[20] Li H.G., Gao H.J., Liu F.F., Liu J. (2017). Quercitrin suppresses hepatocellular carcinoma metastasis and angiogenesis by targeting the Nrf2 signaling pathway. Oncotarget.

[21] Hong C.-O., Lee H.A., Rhee C.H., Choung S.-Y., Lee K.-W. (2013). Separation of the antioxidant compound quercitrin from *Lindera obtusiloba* Blume and its antimelanogenic effect on B16F10 melanoma cells. Biosci. Biotechnol. Biochem..

[22] Chen J., Li G., Sun C., Peng F., Yu L., Chen Y., Tan Y., Cao X., Tang Y., Xie X. (2022). Chemistry, pharmacokinetics, pharmacological activities, and toxicity of Quercitrin. Phytother. Res..

[23] Ahn S.-Y., Lee S.H., Park J.Y., Kim Y.H., Kim J.Y., Kim S.Y. (2024). Potential skin anti-aging effects of main phenolic compounds, tremulacin and tremuloidin, from *Salix chaenomeloides* leaves on TNF-α-stimulated human dermal fibroblasts. Chem.-Biol. Interact..

[24] Choi Y.J., Alishir A., Jang T., Kang K.S., Lee S., Kim K.H. (2022). Antiskin aging effects of indole alkaloid N-glycoside from *Ginkgo biloba* fruit on TNF-α-exposed human dermal fibroblasts. J. Agric. Food Chem..

[25] Pang L.Y., Hurst E.A., Argyle D.J. (2016). Cyclooxygenase-2: A role in cancer stem cell survival and repopulation of cancer cells during therapy. Stem Cells Int..

[26] Wlaschek M., Tantcheva-Poór I., Naderi L., Ma W., Schneider L.A., Razi-Wolf Z., Schüller J., Scharffetter-Kochanek K. (2001). Solar UV irradiation and dermal photoaging. J. Photochem. Photobiol. B Biol..

[27] Shindo Y., Hashimoto T. (1997). Antioxidant defense mechanisms of the skin. Dermatol. Clin..

[28] Fisher G.J., Datta S.C., Talwar H.S., Wang Z.Q., Varani J., Kang S., Voorhees J.J. (1996). Molecular basis of sun-induced premature skin ageing and retinoid antagonism. Nature.

[29] Umesalma S., Sudhandiran G. (2011). Differential inhibitory effects of the polyphenol ellagic acid on inflammatory mediators NF-κB, iNOS, COX-2, and proinflammatory cytokines in colon carcinogenesis. Food Chem. Toxicol..

[30] Kang K.A., Wang Z.H., Zhang R., Piao M.J., Kim H.S., Park B.J., Hyun J.W. (2006). Cytoprotective effect of ellagic acid on hydrogen peroxide-induced oxidative stress in human lung fibroblasts. Biol. Pharm. Bull..

[31] Quan T., Qin Z., Xia W., Shao Y., Voorhees J.J., Fisher G.J. (2009). Matrix-degrading metalloproteinases in photoaging. J. Investig. Dermatol. Symp. Proc..

[32] Zhang H.M., Zhao L., Li H., Xu H., Chen W., Tao L., Shen J., Zheng Y. (2017). Effects of natural products on the regulation of inflammatory pathways in aging. Ageing Res. Rev..

[33] Sies H., Jones D.P. (2020). Reactive oxygen species (ROS) as pleiotropic physiological signalling agents. Nat. Rev. Mol. Cell Biol..

[34] Kim J.H., Lee S.Y., Kim Y.S., Choi H.G., Jang Y.P., Cho H.J. (2010). Ellagic acid attenuates interleukin-1β-induced inflammatory responses in human gingival fibroblasts. Arch. Oral Biol..

[35] Li Y., Liu J., Liu Y., Wang L., Zhang Y. (2006). Biphasic effects of ellagic acid on cell proliferation and apoptosis in human leukemia HL-60 cells. Food Chem. Toxicol..

[36] Junyaprasert V.B., Singhsa P., Suksiriworapong J., Chantasart D. (2012). Physicochemical properties and skin permeation of Span 60/Tween 60 niosomes of ellagic acid. Int. J. Pharm..

[37] Fan G., Xu Z., Liu X. (2017). Preparation of pomegranate Ellagic acid inclusion complex gel and its transdermal permeation in vitro. Procedia Eng..

[38] Mukherjee P.K., Maity N., Nema N.K., Sarkar B.K. (2011). Bioactive compounds from natural resources against skin aging. Phytomedicine.

