# Supplementary material for: Phytochemical Constituents from Cercidiphyllum japonicum Exhibit Bioactive Potential Against Skin Aging and Inflammation in Human Dermal Fibroblasts
Source: Curr Issues Mol Biol. 2025 Aug 7;47(8):631. doi: 10.3390/cimb47080631 (PMC12384587; doi:10.3390/cimb47080631)
Supplement: Supplementary file 1 [file cimb-47-00631-s001.zip › cimb-3761304-supplementary.pdf]

current issues in molecular biology

MDPI

Supplementary material

Ellagic Acid from *Cercidiphyllum japonicum* as a Bioactive Agent Against Skin Aging and Inflammation in Human Dermal Fibroblasts

Minseo Kang [1], Sanghyun Lee [2,3], Dae Sik Jang[4], Sullim Lee[1,]* and Daeyoung Kim [1,]*

[1] Department of Life science, College of Bio-Nano Technology, Gachon University, Seongnam 13120, Republic of Korea; bana825@gachon.ac.kr (M.K.); sullimlee@gachon.ac.kr (Sullim L.); davekim@gachon.ac.kr (D.K.)
[2] Department of Plant Science and Technology, Chung-Ang University, Anseong 17546, Republic of Korea; slee@cau.ac.kr (S.H.L.)
[3] Natural Product Institute of Science and Technology, Anseong 17546, Republic of Korea
[4] College of Pharmacy, Kyung Hee University, 26 Kyungheedae-ro, Dongdaemun-gu, Seoul 02453, Republic of Korea; dsjang@khu.ac.kr (D.S.J.)

* Correspondence: Sullim Lee: sullimlee@gachon.ac.kr; Tel.: +82-31-750-8573; Daeyoung Kim: davekim@gachon.ac.kr; Tel.: +82-31-750-4761

Supplementary Table S1. Instrumentation and Chromatographic Conditions for HPLC Analysis

HPLC	Waters Alliance e2695 Separations Module, USA<br>Waters 2998 Photodiode Array Detector, USA
Column	INNO C18 column (4.6 × 250 mm, 5 μm)
Temperature	30°C
Injection volume	10 μL
Mobile phase	Gradient [A, Water (0.1% TFA) : B, ACN]
Flow	1.0 mL/min
Wavelength	254 nm

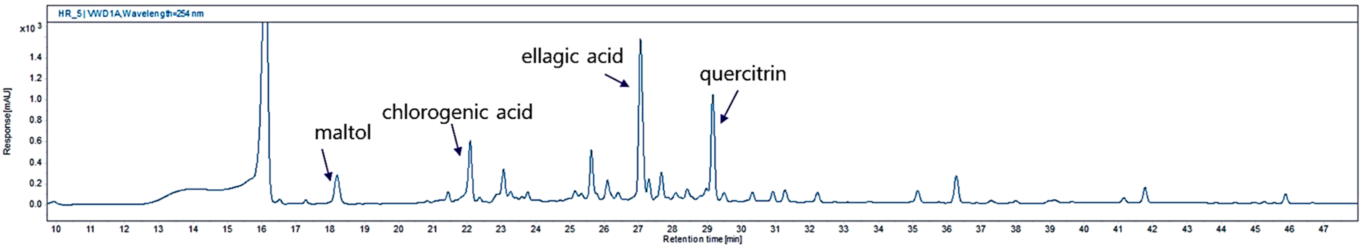

Supplementary Figure S1. HPLC chromatogram of *Cercidiphyllum japonicum* extract detected at 254 nm. Major peaks corresponding to maltol, chlorogenic acid, ellagic acid, and quercitrin are indicated.

 https://doi.org/10.3390/cimb47080631

Supplementary Table S2. Quantification of Major Compounds in Cercidiphyllum japonicum Extract by HPLC Analysis

Compound	t_R	Calibration equation	Correlation factor, r^2	Content (mg/g)
Maltol	18.1	Y = 19.295X + 6.3309	0.9999	4.68 ± 0.03
Chlorogenic acid	22.0	Y = 8.7066X + 108.21	0.9999	17.83 ± 0.65
Ellagic acid	26.9	Y = 72.131X + 623.91	0.9999	5.03 ± 0.02
Quercitrin	29.1	Y = 25.422X + 183.86	0.9998	2.70 ± 0.04
